# Inhibition of Angiotensin-Converting Enzyme Activity by Flavonoids: Structure-Activity Relationship Studies

**DOI:** 10.1371/journal.pone.0049493

**Published:** 2012-11-21

**Authors:** Ligia Guerrero, Julián Castillo, Mar Quiñones, Santiago Garcia-Vallvé, Lluis Arola, Gerard Pujadas, Begoña Muguerza

**Affiliations:** 1 Department of Biochemistry and Biotechnology, Rovira i Virgili University, Tarragona, Spain; 2 Department of Research, Nutrition and Innovation, ALPINA S.A, Bogotá, Colombia; 3 Department of Research and Development, Nutrafur S.A., Murcia, Spain; 4 Centre Tecnològic de Nutrició i Salut (CTNS), TECNIO, CEICS, Avinguda Universitat, Reus, Catalonia, Spain; The University of Manchester, United Kingdom

## Abstract

Previous studies have demonstrated that certain flavonoids can have an inhibitory effect on angiotensin-converting enzyme (ACE) activity, which plays a key role in the regulation of arterial blood pressure. In the present study, 17 flavonoids belonging to five structural subtypes were evaluated *in vitro* for their ability to inhibit ACE in order to establish the structural basis of their bioactivity. The ACE inhibitory (ACEI) activity of these 17 flavonoids was determined by fluorimetric method at two concentrations (500 µM and 100 µM). Their inhibitory potencies ranged from 17 to 95% at 500 µM and from 0 to 57% at 100 *µ*M. In both cases, the highest ACEI activity was obtained for luteolin. Following the determination of ACEI activity, the flavonoids with higher ACEI activity (*i.e.*, ACEI >60% at 500 µM) were selected for further IC_50_ determination. The IC_50_ values for luteolin, quercetin, rutin, kaempferol, rhoifolin and apigenin K were 23, 43, 64, 178, 183 and 196 µM, respectively. Our results suggest that flavonoids are an excellent source of functional antihypertensive products. Furthermore, our structure-activity relationship studies show that the combination of sub-structures on the flavonoid skeleton that increase ACEI activity is made up of the following elements: (a) the catechol group in the B-ring, (b) the double bond between C2 and C3 at the C-ring, and (c) the cetone group in C4 at the C-ring. Protein-ligand docking studies are used to understand the molecular basis for these results.

## Introduction

Cardiovascular disease (CVD) is the most important cause of death among the industrialized societies [1]. Hypertension, which is estimated to affect one-third of the Western population, is one of the major risk factors for CVD [2]. In spite of its significance, hypertension remains poorly controlled [3], and approximately two-thirds of hypertension is undetected or inadequately treated [4]. Lifestyle modifications, including changes in dietary habits, have substantial effects on risk factors for CVD, such as hypertension [5].

The renin–angiotensin–aldosterone system is a key factor in the maintenance of arterial blood pressure. One of its main components is the angiotensin-converting enzyme (ACE) [EC 3.4.15.1] [6], which is a glycosylated zinc dipeptidyl-carboxypeptidase whose main function is to regulate arterial blood pressure and electrolyte balance through the renin–angiotensin–aldosterone system [7]. There are two isoforms of ACE that are transcribed from the same gene in a tissue-specific manner. In somatic tissues, ACE exists as a glycoprotein composed of a single large polypeptide chain of 1,277 amino acids, whereas in sperm cells, it occurs as a lower-molecular-mass glycoform of 701 amino acids. The somatic form consists of two homologous domains (the N and C domains), each of which contains an active site with a conserved HEXXH zinc-binding motif [8], where the Zn^2+^ is bound to the two motif histidines as well as to a glutamate 24 residue downstream the last motif histidine [9]. The testis ACE (tACE) is identical to the C-terminal half of somatic ACE, with the exception of a unique 36-residue sequence that constitutes its amino terminus [10]. The two domains differ in their substrate specificities, inhibitor and chloride activation profiles, and physiological functions [11]. Thus, mice expressing only the N domain of ACE show a low blood pressure phenotype that is very similar to ACE knockout mice [12], and ACE inhibition with an N-domain-specific inhibitor (*i.e.*, RXP407) has no effect on blood pressure regulation [13]. On the other hand, mice that are homozygous for a mutation that inactivates the somatic ACE N domain, but not the C domain, retained a phenotype that was indistinguishable from that of wild-type mice with regards to blood pressure and renal function [14]. Therefore, the inhibition of the C domain appears to be necessary and sufficient for the control of blood pressure and cardiovascular function, which suggests that the C domain is the dominant angiotensin-converting site. As an exopeptidase, ACE catalyzes the conversion of angiotensin I into the potent vasoconstrictor angiotensin II [15]. In addition, ACE catalyzes the inactivation of the vasodilator bradykinin [16]. Therefore, the inhibition of this enzyme can generate an antihypertensive effect. In fact, synthetic ACE inhibitors, such as captopril and enalapril, are widely used for the treatment of cardiovascular and renal disease, for the secondary prevention of coronary artery disease, and for the treatment of heart failure [17]. However, side effects such as cough, angioneurotic edema and deleterious effects in pregnancy have been associated with the clinical use of ACE inhibitors [18], [19]. Therefore, the investigation of new, natural product-based ACE inhibitors could greatly benefit hypertensive patients.

**Table 1:** HPLC analysis of the flavonoid samples used in the current study.

		Main content of the minor flavonoids	
Compound	content (%)^1^	(%)^2^	Other minor flavonoids
**Naringenin**	94.7	Naringin (0.6)	Naringenin-7-glucoside
**Naringenin K**	93.8	Naringin (0.9)	Naringenin-7-glucoside
**Naringin**	95.2	Narirutin (1.1)	Poncirin, Naringenin
**Apigenin**	97.2	Rhoifolin (0.7)	Apigenin-7-glucoside
**Apigenin K**	96.5	Rhoifolin (0.8)	Apigenin-7-glucoside
**Rhoifolin**	96.1	Naringin (0.4)	Apigenin
**Genistein**	97.2	Daidzein (1.1)	Genistin
**Luteolin**	95.9	Eriodictyol (0.8)	Luteolin-7-glucoside
**Hesperetin**	94.7	Hesperidin (1.2)	Hesperetin-7-glucoside
**Diosmetin**	93.8	Diosmin (2)	Hesperidin
**Diosmin**	95.2	Hesperidin (1.8)	Hesperetin
**Catechin**	98.1		Other catechins
**Epicatechin**	98.5		Other catechins
**Quercetin**	95.7	Rutin (1.5)	Isoquercitrin
**Rutin**	97.1	Isoquercitrin (1.1)	Quercetin
**Kaempferol**	96.8	Quercetin (0.8)	Kaempferol-3-glucoside

1 Absolute value as is.

2 The reference % assay is referred to the absolute content as is of the main flavonoid.

A number of extracts and compounds obtained from plants have been identified as *in vitro* ACE inhibitors [20], [21]. These beneficial effects have largely been ascribed to the presence of flavonoid molecules, which generation of chelate complexes within the active center of ACE [22]. Flavonoids are polyphenol molecules of low molecular weight; the basic structure is a 2-phenyl benzopyrone in which the three-carbon bridge between the phenyl groups is usually cyclized oxygen [23], [24]. Flavonoids can be differentiated into several subfamilies according to their degree of unsaturation and the degree of oxidation of the oxygenated heterocycle and can be characterized as flavanones, flavones, flavonols, isoflavones, flavanols (essentially, flavan-3-ols) and anthocyanidins, all of which are the most relevant for the human diet [23], [25]. Different studies have revealed the important role that flavonoid structure plays in its biological function; the position and number of substituents in the flavonoid basic structure significantly affects the antiproliferative, cytotoxic, antioxidant, and anti-enzymatic activities of such molecules [26]–[28].

**Figure 1:**
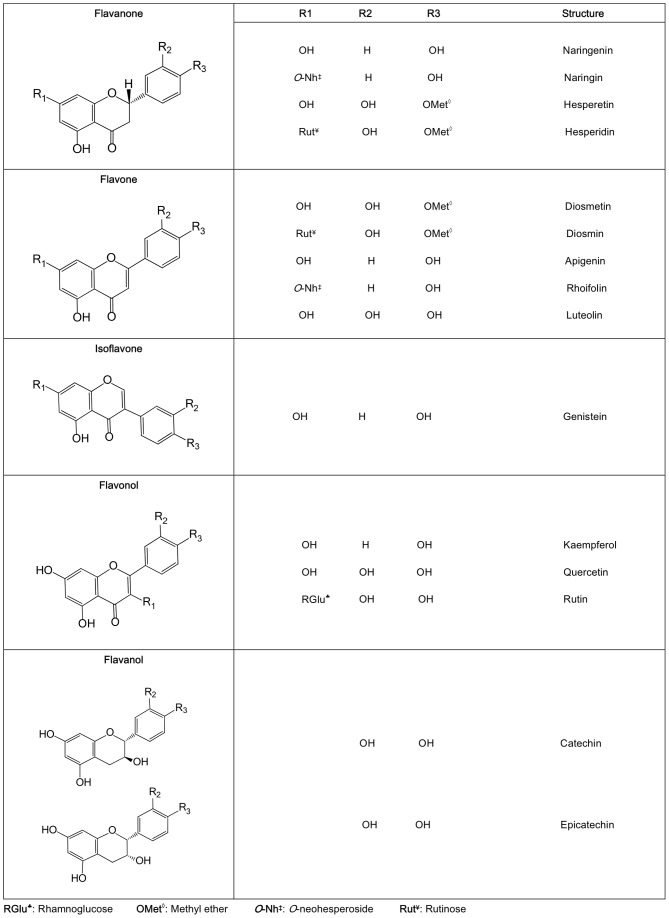
Structures of the different flavonoids used in this study.

Previous studies have shown that certain flavonoids exhibit a capacity to inhibit different zinc metalloproteinases [29], [30], including ACE. In fact, micromolar concentrations of different flavonoids, such as anthocyanins [31], [32], flavones [33], flavonols [33]–[35], and flavanols [36], have been shown to inhibit 50% of ACE activity. Furthermore, the ACE-inhibitory (ACEI) activity of different foods and plant extracts rich in flavonoids has also been demonstrated by *in vitro* [37], [38], studies and by *in vivo* studies in hypertensive rats [39], [40] and humans [41]. The preliminary structure-activity relationships (SAR) studied in some flavonoids (flavanols and flavonols) generally attribute the observed effect either to the distribution of free hydroxyl groups [33], [35], [42] or in the number of monomers units forming the corresponding procyanidins [36]. However, the key molecular flavonoid sub-structures that dictate effective ACE inhibition activity have not yet been characterized.

**Figure 2:**
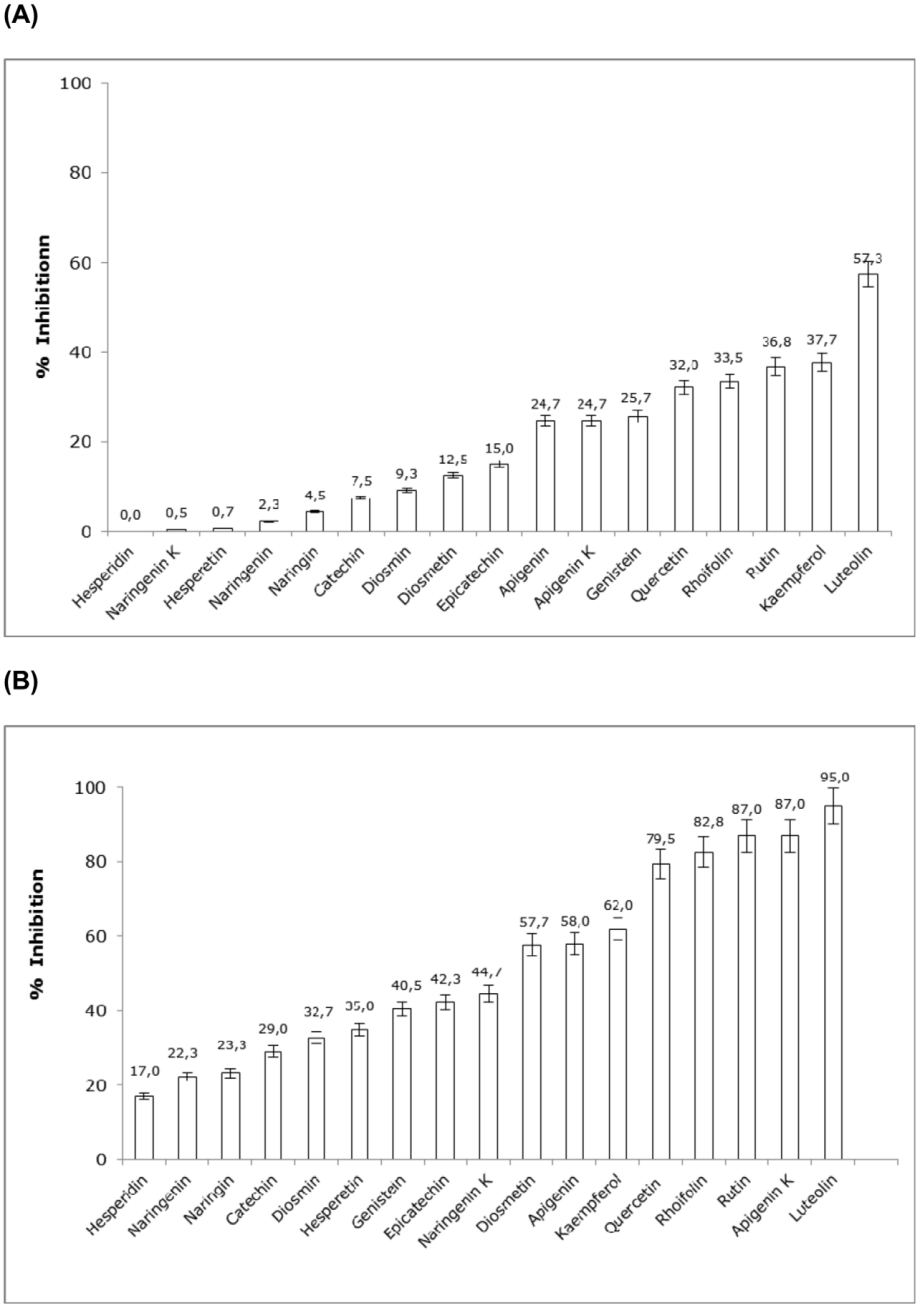
(A) Effect of different flavonoids on Angiotensin Converting Enzyme (ACE) activity. Purified lung ACE was preincubated at 37°C for 30 min in the presence of 100 µM of flavonoids or DMSO as a control. The results are expressed as the percentage of ACE inhibition. The plot represents the mean result ± SD from three experiments. (**B**) Effect of different flavonoids on Angiotensin Converting Enzyme (ACE) activity. Purified lung ACE was preincubated at 37°C for 30 min in the presence of 500 µM of flavonoids or DMSO as control. The results are expressed as the percentage of ACE inhibition. The plot represents the mean ± SD from three experiments.

**Table 2:** IC_50_ values obtained for the selected flavonoids.

	IC_50_ value^1^ (µM)
Apigenin K	196
Rhoifolin (apigenin 7-O-glycoside)	183
Kaempferol	178
Rutin (quercetin 3-O-glycoside)	64
Quercetin	43
Luteolin	23

1 The IC_50_ value represents the concentration of each compound that inhibits ACE activity by 50%.

The objective of this work was to define the key flavonoid structural elements that are required for ACE inhibition activity through the determination of the ability of 17 flavonoids belonging to five structural subtypes (*i.e.*, 5 flavanones, 2 flavan-3-ols, 1 isoflavone, 6 flavones and 3 flavonols; including potassium salts for 1 flavanone and for 1 flavone) to inhibit ACE. To achieve this goal, the *in vitro* ACE inhibition activity of these 17 flavonoids was measured, and the corresponding results were used to establish SAR for these molecules. Afterwards, protein-ligand docking studies were used to describe the molecular basis for most significant SAR results.

**Figure 3:**
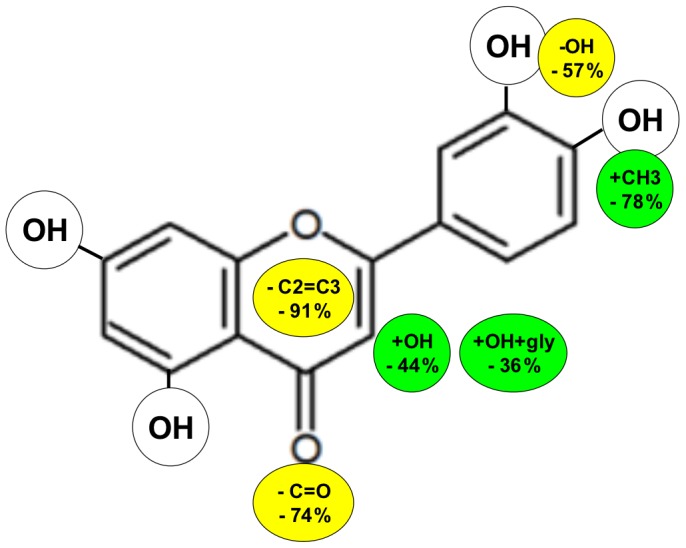
Structural diagram which quantitatively assesses the effect of the addition or elimination of different structural elements from the flavonoid core on the ACEI activity of luteolin at 100 µM. According to these data, the significance order was: double bond C2 = C3 (absence: −91% activity by comparing naringenin *vs.* apigenin) >4′-O-methoxylation (presence: −78% by comparing diosmetin *vs.* luteolin) ≈ 4-carbonyl group (absence: −74% by comparing epicatechin *vs.* luteolin) >3′-hydroxylation (absence: −57% by comparing apigenin *vs.* luteolin) >3-hydroxylation (presence: −44% by comparing quercetin *vs.* luteolin) >3-O-glycosylation (presence: -36% by comparing rutin *vs.* luteolin).

## Materials and Methods

### Chemicals

*o*-aminobenzoylglycyl-*p*-nitro-phenylalanylproline (*o*-ABz-Gly-Phe(NO_2_)-Pro) was purchased from Bachem Feinchemikalien (Bubendorf, Switzerland). Five units of Angiotensin-I Converting Enzyme from rabbit lung and ZnCl_2_ were obtained from Sigma (Barcelona, Spain). All flavonoids (assay >90% purity) used in this study were kindly provided by Nutrafur S.A. (Murcia, Spain), except for the catechin, luteolin and genistein, which came from Sigma-Aldrich Química (Barcelona, Spain). Flavonoids were solubilized in dimethyl sulfoxide (DMSO) and prepared daily. In all experiments, the final concentration of DMSO was 0.4%. Distilled water was obtained from a Millipore Milli-Q® system.

**Table 3:** Intermolecular interactions between ACE inhibitors and the tACE binding site.

		LISINOPRIL	ENALAPRILAT	CAPTOPRIL	RXPA380	SELENOCAPTOPRIL	KAF	KAW	lisW-S	FII-A	LUTEOLIN	QUERCETIN	RUTIN	KAEMPFEROL
**S2**′	Gln281	✓	✓	NE2	NE2	NE2	NE2	✓	H	H				
	Thr282										✓	✓	✓	✓
	His353	NE2	NE2	NE2	NE2	NE2	NE2	NE2	NE2	NE2	✓	✓	✓	✓
	Glu376									✓			✓	
	Asp453													✓
	Lys511	NZ	NZ	NZ	NZ	NZ	NZ	NZ	NZ	NZ				
	His513	NE2	NE2	NE2	NE2	NE2	NE2	NE2	NE2	NE2				
	Tyr520	OH	OH	OH	OH	OH	OH	OH	OH	OH				
**S2**′**/S1**′	Ser284													
	Val379										✓	✓	✓	✓
	Val380	✓							✓	✓	✓		✓	✓
**S1**′	Glu162	✓											OE2	
	Asn277												✓	
	Asn374													
	Asp377												OD1	
**S1**	Glu143													
	Val351													
	Ala354	O	O	✓	✓	✓	✓	✓	O	✓	✓	✓	O	O
	Ser355		✓		✓		✓	✓		✓	✓	✓	✓	
	Trp357													
	Lys368													
	Glu384	OE2	OE2	OE2	OE2	✓	OE2	OE2	OE2	OE2	✓	✓	✓	
	Phe512	✓	✓		✓		✓	✓	✓	✓				
	Ser516													
	Tyr523	OH	OH	✓	OH	✓	OH	OH	OH	OH	OH	OH	OH	✓
**S1/S2**	Val518	✓	✓		✓		✓	✓	✓	✓				
**S2**	Phe391				✓		✓	✓		✓				
	Glu403													
	Arg522													
**Other**	Thr166												✓	
	Trp279												✓	
	Ala356				N		N	N		N				
	His383	✓	✓	✓	NE2	✓	NE2	NE2	NE2	NE2				✓
	His387	NE2	✓	✓	NE2		✓	✓	NE2	NE2				
	His410				✓		✓			✓				
	Glu411	✓	✓		✓		OE1	✓	✓				OE1	
	Asp415				✓			✓	OD2		✓	OD1	✓	✓
	Lys454												NZ	NZ
	Phe457	✓	✓	✓	✓	✓	✓	✓	✓	✓				
	Phe527								✓	✓			✓	

Data used for lisinopril, enalaprilat, captopril, RXPA380, selenocaptopril, KAF, KAW, lisW-S and FII-A was obtained from the LigPlot+ diagrams that are available at the PDBsum resource (http://www.ebi.ac.uk/pdbsum/) for PDB files 1O86, 1UZE, 1UZF, 2OC2, 2YDM, 3BKK, 3BKL, 3L3N and 2XY9, respectively. Data for luteolin, quercetin, rutin and kaempferol was obtained by applying LigPlot+ to the structure of their predicted complexes with tACE. Hydrophobic contacts are indicated by a check mark whereas hydrogen bonds are indicated with the label of the protein atom that is involved.

### Preparation of Solutions

The 0.45 mM buffered substrate solution (*o*-Abz-Gly-p-Phe(NO_2_)-Pro) and 150 mM Tris-acid buffer solution containing 1.125 M NaCl (pH 8.3) were prepared daily. Flavonoid solutions (100 and 500 µM) were prepared in DMSO (0.4%) daily. The 0.1 U/mL ACE solution stock was prepared in glycerol: water (1∶1), aliquoted and stored at −20°C. The 0.1 µM ZnCl_2_ stock solution was prepared and stored at 4°C. The ACE working solution was prepared daily by diluting it in 150 mM Tris buffer (pH 8.3) containing 0.1 µM ZnCl_2_.

**Figure 4:**
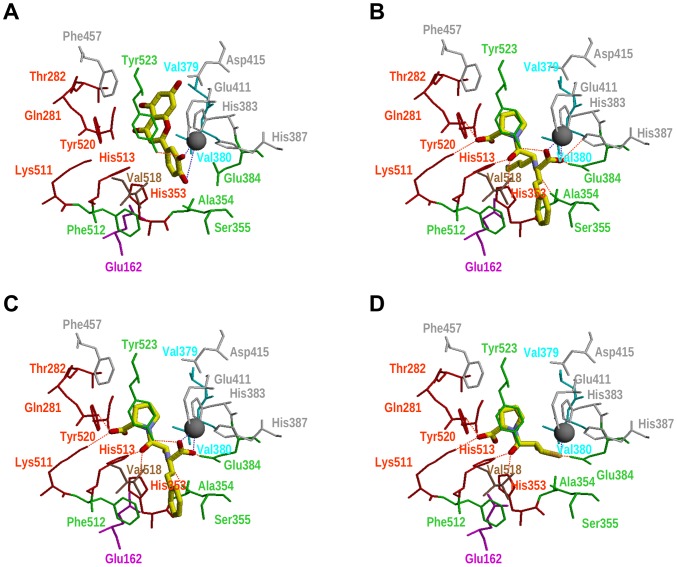
Best predicted pose for luteolin (panel A) at the tACE binding site and the relative location to experimental poses for the ACE inhibitors lisinopril (panel B), enalaprilat (panel C), and captopril (panel D). All of the panels in this figure are in the same relative orientation to allow for easier comparisons between the poses. Residues at the ACE binding site are colored according to the subsite where they belong (*i.e.*, residues from the S2′, S2′/S1′, S1′, S1 and S1/S2 subsites are colored in red, cyan, magenta, green, brown, white and yellow, respectively). Other important residues that have not been classified in any pocket are colored in white. Carbon atoms for the ligands are shown in yellow to make them more easily distinguishable from the binding site residues. Dashed lines are used to show intermolecular hydrogen bonds (in red) or charge-charge interactions (in blue).

### Chromatographic Analysis and Quantification of Flavonoid Compounds

For the elucidation and quantification of the main flavonoids present in each sample, we modified a previously published method [27]. All the samples were dissolved in DMSO in the ratio of 5 mg/mL, and the resulting solutions were filtered through a 0.45 µm nylon membrane. The HPLC equipment was a Hewlett-Packard Series HP 1100 equipped with a diode array detector. The stationary phase was a C18 LiChrospher 100 analytical column (250 × 4 mm i.d.) with a particle size of 5 µm (Merck, Darmstadt, Germany) thermostated at 30°C. The flow rate was 1 mL/min and the absorbance changes were simultaneously monitored at 280 and 340 nm. The mobile phases for chromatographic analysis were: (A) acetic acid: water (2.5∶97.5) and (B) acetonitrile. A linear gradient was run from 95% (A) and 5% (B) to 75% (A) and 25% (B) for 20 min; changed to 50% (A) and (B) for 20 min (40 min, total time); changed to 20% (A) and 80% (B) for 10 min (50 min, total time), and finally re-equilibrated for 10 min (60 min, total time) to the initial composition. Table 1 resumes the global HPLC profile of the different samples used in this study.

### Measurement of ACE-inhibitory Activity

ACEI activity was measured by a fluorimetric assay following the method of Sentandreu and Toldrá [43] with some modifications [44]. A volume of 160 µL of 0.45 mM buffered substrate solution in 150 mM Tris-acid buffer containing 1.125 M NaCl, (pH 8.3) was mixed with 40 µL of the flavonoid solution (with 0.4% DMSO for the blank samples) and 40 µL of ACE solution (0.1 U/mL), and the mixture was incubated at 37°C. Fluorescence was measured after 30 min in 96-well microplates (Thermo Scientific, Rochester, NY) using a multiscan microplate fluorimeter (Biotek. FL×800). Microplates (Thermo Scientific, Rochester, NY) were used in this assay. The excitation and emission wavelengths were 360 and 430 nm, respectively. The activity of each sample was tested in technical and biological triplicate.

The ACEI activity was calculated using the following formula:
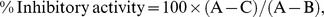
where A is the fluorescence without the flavonoid solution, B is the fluorescence without ACE and C is the fluorescence in the presence of both ACE and the flavonoid solution. A flavonoid solution of 500 µM was selected on the basis of previous studies [33], [35], and the concentration of 100 µM was chosen because it is within the physiological concentration range. An ACEI activity higher than 60% at 500 µM concentration was used as a selection criterion for the IC_50_ (the flavonoid concentration required to inhibit the original ACE activity by 50%). The IC_50_ of each selected flavonoid was tested in technical and biological triplicate. The results from three experiments are expressed as the mean ± SD and were performed in different platelet samples.

### Molecular Modeling Studies

Flavonoid structures were either obtained from ChemSpider (http://www.chemspider.com/) or drawn with Marvin Sketch v5.9.0 (ChemAxon Kft., Budapest, Hungary; http://www.chemaxon.com/). All flavonoid structures were further set up with LigPrep v2.5 (Schrödinger LLC, Portland, USA; http://www.schrodinger.com) following three steps: **(1)** using Epik software [45] to generate all possible protonation states within a pH range of 4.0±7.0 and selecting the “add metal binding states” option to generate possible ligand-metal binding states among metalloproteins; **(2)** generating tautomers at the previously given pH range; and **(3)** determining chiralities from the ligand’s 3D structure.

All the protein-ligand docking studies performed in this work were performed with Glide v5.7 (Schrödinger LLC., Portland, USA; http://www.schrodinger.com) [46], [47] with extra precision (*i.e.*, with GlideXP; [48]). Before docking the flavonoids, the shape and properties of the ACE binding site were represented by several different sets of fields on a grid. This grid was made of a box that has default dimensions around the location of the experimental pose of the inhibitor (*i.e.*, RXPA380), and the inhibitor forms a complex with tACE at PDB file 2OC2 [49]. No constraints were set while building the grid. Default settings for the rest of the grid set-up options were used. During the protein-ligand docking, a maximum number of 5 poses per ligand were obtained. Then, the most reliable pose was selected (irrespective of its glide score) by taking advantage of the information provided by **(1)** the other experimental complexes between ACE and ACE inhibitors available in the PDB (Protein Cate Base) (http://www.pdb.org) and **(2)** the SAR results obtained in the current study for flavonoids. No constraints were imposed during the docking except for those flavonoids in which all docked poses were far from the area predicted for either luteolin or quercetin. In those cases, the AC ring location of their selected poses was used to restrict their docking (*i.e.*, luteolin was used during apigenin and diosmetin docking, whereas quercetin was used to restrict the docking of rutin and kaempferol). Restricted docking with rhoifolin failed as a consequence of the steric hindrance between the ACE binding site and the 7-O-glycoside substituent; therefore, no docking results are reported for rhoifolin. The results for predicted poses were compared with known experimental poses for ACE inhibitors by comparing their intermolecular interactions with the ACE binding site. With that aim, LigPlot diagrams for the experimental ACE-inhibitor complexes were obtained from the PDBsum website (http://www.ebi.ac.uk/pdbsum/) and compared with equivalent diagrams derived from LigPlot+ [50] for predicted ACE-flavonoid complexes.

## Results and Discussion

Seventeen flavonoids were evaluated for their ACEI activity. The structures of all compounds studied are represented in Figure 1. All the flavonoids were studied at concentrations of 100 and 500 *µ*M (see Figures 2A and 2B, respectively). The maximum inhibitory potencies were 57% at 100 *µ*M and 95% at 500 *µ*M. At both concentrations, the highest ACEI activity was exhibited by luteolin. The relative inhibitory potencies for the most active flavonoids (*i.e.*, ACEI higher than 30%) were luteolin>apigenin K>rutin>rhoifolin>quercetin>kaempferol>apigenin>diosmetin>narigenin K>epicatechin>genistein>hesperetin and diosmin for 500 *µ*M; and luteolin>kaempferol>rutin>rhoifolin>quercetin for 100 *µ*M. The rest of the flavonoids exhibited ACEI activities lower than 30%. The IC_50_ value was obtained for each flavonoid that exhibited an ACEI activity higher than 60% at 500 µM (*i.e.*, luteolin, apigenin K, rutin, rhoifolin, quercetin and kaempferol; see Table 2). These IC_50_ values were found to be in the 23 to 196 *µ*M range (with luteolin being the flavonoid with the highest ability to inhibit ACE activity).

In recent years, flavonoids have gained a great amount of interest with regards to their potential for cardiovascular protection. In fact, many epidemiological studies associate an increased consumption of foods and beverages rich in flavonoids with a reduced risk of CVD death [51]–[53]. Additionally, several of these flavonoids or their derivatives (*i.e.*, diosmin, rutin and quercetin) are widely used as pharmaceutical agents for their vasoprotective properties (*i.e.*, Daflon 500 and Venorutom) [54].

Flavonoids are based on the structure of phenyl-benzopyrone and differ from one another in terms of hydroxyl, methoxyl or glycosylated substituents, the position of the benzenoide (B-ring) substituent relative to the C-ring, the degree of unsaturation and the types of sugars that are attached [55]. We evaluated the inhibitory effects on the ACE activity of a group of flavonoids from five different structural types (see Figure 1). The inhibitory effects of certain flavonoids on ACE activity that have been reported in other studies were confirmed [37]. Many of the flavonoids that were tested could inhibit ACE in the micromolar range [22], [56]. However, as was expected, significant differences were observed in the ACEI activity depending on the flavonoid structure [25], [36]. Although the ACEI activity of these flavonoids does not reach the potency of drugs commonly used in the treatment of hypertension, food products with moderate ACEI activity (*i.e.*, an ACEI index higher than 70%) may be considered as naturally functional foods [57] if it is also taken into account that the regular dietary intake of polyphenols could be as high as 1 g/day [58], [59]. Moreover, functional foods containing these natural compounds would not be expected to have the side effects associated with synthetic drugs used in hypertension control [60].

Our evaluation of the abilities of different flavonoids to inhibit the activity of ACE confirmed that the principal structural features for their inhibitory activity are as follows: (a) the double bond between C2 and C3 at the C-ring; (b) the catechol group in the B-ring (3′,4′-dihydroxy) [61]; and (c) the cetone group at the C4 carbon on the C-ring [which is a functional group that has been observed to be essential for inhibiting ACE [62]. According to these general considerations, we analyzed and evaluated the SAR derived from our results. Our data confirm that a distinguishing feature for ACE inhibition by flavonoids is the presence of an unsaturated 2–3 bond conjugated with a 4-oxo- function, aside from the 3′,4′-catechol B-ring pattern, as occurs mainly in luteolin, quercetin and rutin. However, it is important to analyze the specific, qualitative and quantitative influence of each one of these three sub-structures in the SAR results.

### The Significance of the C2 = C3 Double Bond in the C-ring: Flavone *vs.* Flavanone

As previously mentioned, the presence of a C2–C3 double bond seems to be essential for the molecule to exert a significant ACEI activity. Two main factors would explain this fact. First, the molecular electronic distribution would allow the maintenance of a definitive structural conjugation, from the B-ring to the A and C rings, in contrast to the flavanone structure, with which this definitive structural conjugation is not possible. Second, the spatial, or the maintenance of a nearly planar structure, would disappear if this bond was saturated, producing a flavonoid skeleton with an obtuse angle, which would be variable depending on the rest of the constituents of the molecule. Our data confirm previous findings that suggest that a nearly planar flavonoid structure is an important factor in the inhibition of ACE [55]. In fact, all flavanones included in this study, both aglycones (naringenin and hesperetin) and glycosides (naringin), are not as effective as flavones on ACEI activity. This difference can be observed, more specifically, by comparing the results between the flavone apigenin and its corresponding structurally similar flavanone, naringenin (and, although the difference is on another scale, between diosmetin and hesperetin) where the absence of the C2 = C3 double bond in naringenin involves a 91% reduction of ACEI activity at 100 µM *vs.* apigenin (see Figures 2A and 3).

### B-ring Pattern: Catechol Group *vs.* Monohydroxy Group and O-methylation

The presence of several hydroxyl groups in the flavonoids seems be important for the extent of inhibition of the zinc metalloproteinases [29]. Additionally, the exact position of this group has been revealed to be very important for ACE inhibition. Hydroxylation at the 4′-position of the B ring seems to be of particular relevance, and in addition, the presence of a catechol group in the B ring (3′,4′-dihydroxy) appears to be fundamental to achieving an increased ACE inhibitory activity, as occurred in luteolin (as well as quercetin and rutin), which presented the highest ACEI efficiency (see Figure 2 and Table 2). Luteolin has also been described as the most effective flavonoid for inhibiting other metalloproteinases (aminopeptidases), such as MMP-1 and MMP-2 [30]. Consequently, the presence of a catechol at the B-ring should be considered to be very important; indeed, the absence of the 3′ hydroxyl group in apigenin causes a 57% reduction of ACEI activity at 100 µM relative to the luteolin (see Figures 2A and 3). A similar reduction of activity occurs with the flavonols quercetin and kaempferol, where the absence of catechol in the kaempferol resulted in a 4-fold increase in the IC_50_ relative to quercetin (see Table 2).

Additionally, the characteristic esterification of flavonoids in the 4′ position significantly reduces ACEI activity, as occurs when the 4′-hydroxyl group of luteolin is methylated to generate diosmetin (an esterification that causes a 78% reduction of ACEI activity at 100 µM; see Figures 2A and 3).

### The Absence of a Carbonyl Group in C4 (C-ring)

The absence of this functional group represents an important reduction in the capacity to inhibit ACE activity. This fact was evident in the case of flavan-3-ols, catechin and epicatechin in comparing their ACEI activities with the inhibition exerted by the luteolin, although all of them have a catechol structure in their B ring. However, it is important to keep in mind that the disappearance of the carbonyl group is simultaneously accompanied by the disappearance of the double bond C2 = C3, the structural significance of which has been previously mentioned. This effect is perhaps related to the loss of planar spatial structure [26], [62].

### The Significance of 3-hydroxylation of the C-ring: Flavone *vs.* Flavonol

According to all the structural aspects mentioned above, the ACEI activity of flavonoids are related to a specific electronic distribution, the minimal alteration of this electronic distribution on the flavonoid skeleton, such as the transformation of flavone into flavonol by the addition of a hydroxyl group in the C-3 position of the C ring, thus, luteolin to quercetin, produced a 44% decrease in the ACEI activity at 100 µM (see Figures 2A and 3). However, a similar reduction in ACE activity does not occur when comparing the activity of apigenin and kaempferol because in this case, the flavonol exerts an increase in the ACEI activity with respect to the flavone at both the tested concentrations. This suggests that the absence of a catechol group in the B ring of apigenin and kaempferol also modifies the global electronic distribution, altering the significance of the hydroxylation at C-3.

### The Significance of Glycosylation at 7-O (A-ring) and 3-O (C-ring) Positions

These two positions are the most typical locations for glycosyl radicals in the flavonoid skeleton (generally, rhamnose-glucose-aglycon). Table 2 shows that the flavonoids rhoifolin (7-O-gly) and rutin (3-O-gly) were almost as effective as their corresponding aglycons (*i.e.*, apigenin and quercetin, respectively) indicating that glycosylations at positions 7 or 3 do not produce steric hindrances that hamper them from binding to ACE in a way that is similar to the actions of their own aglycons.

These data show similarities in activity levels for the naringin-naringenin tests but demonstrate the opposite effects for diosmin-diosmetin (see Figure 2). This behavioral difference could be related to differences in the glycosylation pattern: neohesperidosyl (rha-1-2-glu) for rhoifolin and naringin, and rutinosyl (rha-1–6-glu) for diosmin. The neohesperidosyl structure has greater electronic interaction with the flavonoid skeleton (including intramolecular hydrogen bonds), with consequent influence on the overall electronic distribution. The fact that the 7-O-glucoside flavonoids (glucose-aglycon) were equal or even stronger inhibitors than the aglycons has already been reported with relation to the inhibition of the zinc metalloproteinases MMP-2 and MMP-9 [30].

### The Significance of the Position of B-ring in C-ring: Flavone (2-B-ring) *vs.* Isoflavone (3-B-ring)

The data from this study do not allow a definite assessment of ACEI activity in the tested compounds. Although the values obtained for the isoflavone genistein and the flavone apigenin show differences at 500 µM (40% *vs*. 58%, respectively), the responses were similar at the 100 µM concentration.

### The Possible Significance of Potassium Salts

More research is needed to evaluate the use of flavonoids in which the hydroxyls in positions 7 and 4′ are ionized (R-O-K). At 100 µM, apigenin and naringenin showed similar effects to those of their potassium salts, while at 500 µM, their potassium salts showed a significantly higher ACEI activity.

### IC_50_ Values for Selected Compounds

Independent of the comparative analysis of the results obtained at 100 and 500 µM, the determination of the IC_50_ value for six selected compounds allows us to confirm the importance of the sub-structural elements of the SAR (see Table 2). Thus, the relative IC_50_ ratios of these compounds ([*i.e.*, luteolin (1)>quercetin (1.87)>rutin (2.78)>kaempferol (7.74)>rhoifolin (7.96)>apigenin K (8.52)] confirm the importance of the presence of the B-ring catechol group combined with the double bond and the carbonyl group of the C ring. In fact, the absence of the above-mentioned catechol group for kaempferol, rhoifolin and apigenin supposes an increase of the IC_50_ up to values that are most likely within a range of physiological concentrations. Therefore, to reach the concentration of flavonoids necessary to inhibit the ACE, it would be necessary to apply them as pharmaceuticals agents or as dietary supplements which contain a sufficient flavonoid concentration to obtain *in vivo* efficacy. The apparent influence of 3-O-glycosylation (rutin *vs.* quercetin) would seem to be more logical, in that the existence of a certain steric impediment inhibits the interaction of the flavonoid with the active center of the enzyme, although the values for both flavonols are reasonably similar.

### Prediction of Complexes between tACE and the Flavonoids with the Highest ACEI Activity

The intermolecular interactions between ACE’s binding site residues and the predicted poses for the flavonoids with the highest ACEI activity are shown in Table 3 (together with results for experimental complexes at PDB between tACE and ACE inhibitors). The results in that table show how these flavonoids form intermolecular interactions with some of the ACE’s binding site regions (*i.e.*, S2′, S2′/S1′ and S1) that are also involved in the binding of known ACE inhibitors. At this point, it is remarkable how these flavonoids share most of the intermolecular interactions at the ACE’s S1 site that have been found for synthetic ACE inhibitors (*e.g.*, lisinopril, enalaprilat, captopril; see Table 3) and, therefore, suggest that binding to the ACE’s S1 subsite is essential for ACEI activity.

Figure 4 shows the predicted pose for luteolin at the tACE binding site and its location relative to the experimental poses for the ACE inhibitors lisinopril, captopril and enalaprilat. In that pose, the two B-ring hydroxyls of luteolin are able to make charge-charge interactions with the active site Zn^2+^ (in a way that is similar to the interactions made by the carboxylic acid from lisinopril and enalaprilat; see Figure 4). Interestingly, the charge-charge interaction with the 3′ hydroxyl is stronger than that of with the 4′ interaction (*i.e.*, the distance between the Zn^2+^ ion and the hydroxyl oxygen is 2.1 and 4.3 Å for 3′ and 4′, respectively) which could explain why apigenin (see Figure 1) shows a decrease of approximately 50% of ACE inhibition relative to luteolin (see Figure 2).

### Conclusions

In this study, we have demonstrated that changes in the flavonoid active core affect its capacity to inhibit the ACE, in a way that is similar to what has been described for other zinc metalloproteinases [29], [30]. We provide additional examples of flavonoid structure–activity relationships and establish the structural features needed for the ACEI activity of flavonoids. We show that at the physiological flavonoid concentration (*i.e.*, 100 µM), the relative effect of the different substructures on ACEI activity is as follows: double bond C2 = C3>4′-O-methoxylation ≈ 4-carbonyl group>3′-hydroxylation >3-hydroxylation >3-O-glycosylation. Through this study we can assess the influence of different structural groups, at the steric level, on inductive-mesomeric effects and the flavonoid molecular skeleton. Finally, we would like to remark that it is clear that the application of these flavonoids as inhibitors of ACE *in vivo* may be useful as nutritional supplements or in pharmaceutical formulations to obtain a sufficient dose/response efficacy.
